# The Benefits of Toxicity: *M. smegmatis* VapBC TA Module Is Induced by Tetracycline Exposure and Promotes Survival

**DOI:** 10.3390/microorganisms11122863

**Published:** 2023-11-26

**Authors:** Mikhail Zamakhaev, Julia Bespyatykh, Anna Goncharenko, Mikhail Shumkov

**Affiliations:** 1 Federal Research Center Fundamentals of Biotechnology of the Russian Academy of Sciences, 33, bld. 2 Leninsky Ave., 119071 Moscow, Russia; pylaevanna@gmail.com (A.G.); shumkovm@gmail.com (M.S.); 2 Federal Research and Clinical Center of Physical-Chemical Medicine of Federal Medical Biological Agency, 1A Malaya Pirogovskaya St., 119435 Moscow, Russia; juliabes@rcpcm.org; 3 Expertise Department in Anti-Doping and Drug Control, Mendeleev University of Chemical Technology of Russia, 9, Miusskaya Sq., 125047 Moscow, Russia

**Keywords:** toxin-antotoxin loci, bacterial dormancy, *M. smegmatis*, antibiotic resistance, VapC toxin, MazF toxin

## Abstract

Toxin–antitoxin (TA) systems are widely present in bacterial genomes. *Mycolicibacterium smegmatis*, a common model organism for studying *Mycobacterium tuberculosis* physiology, has eight TA loci, including *mazEF* and *vapBC*. This study aims to investigate the physiological significance of these TA systems. Proteomic profiling was conducted on a culture overexpressing the VapC toxin, and the involvement of VapC in *M. smegmatis* stress responses to heat shock and antibiotic treatment was examined. While deciphering the underlying mechanisms of the altered stress resistance, we assessed the antibiotic susceptibility of *vapBC*, *mazEF*, and double *vapBC-mazEF* deletion mutants. Additionally, the mRNA levels of *vapC* and *mazF* were measured following tetracycline supplementation. The results reveal changes in the abundance of metabolic enzymes and stress response proteins associated with VapC overexpression. This activation of the general stress response leads to reduced thermosensitivity in *M. smegmatis*, but does not affect susceptibility to ciprofloxacin and isoniazid. Under tetracycline treatment, both *vapC* and *mazF* expression levels are increased, and the fate of the cell depends on the interaction between the corresponding TA systems.

## 1. Introduction

Bacterial toxin–antitoxin (TA) loci are genetic elements found in bacterial genomes that consist of a pair of closely linked genes encoding a toxin and an antitoxin [1]. In type II TA systems, the toxin gene encodes a protein that inhibits essential cellular processes, leading to growth arrest or cell death, whereas the antitoxin gene, on the other hand, encodes a protein that neutralizes the toxin’s harmful activity by binding to it [2]. TA modules are widespread and can be localized both on plasmids and in chromosomes of bacterial cells. At least 93 TA loci have been found in *Mycobacterium tuberculosis* (MTB), which is the causative agent of tuberculosis, and more than 50 of them belong to the *vapBC* TA system, whereas 11 are defined as the *mazEF* modules [3,4]. *Mycolicibacterium smegmatis* (basonym *Mycobacterium smegmatis*), which is used often as a non-pathogenic model organism for studying various aspects of mycobacterial biology as it shares many genetic and physiological characteristics with pathogenic mycobacteria species [5], possesses eight TA loci. These include one *mazEF* (*MSMEG_4447*-*4448*) [6] and two *vapBC* (*MSMEG_1283*-*1284* and *MSMEG_6760-6762)* systems, though the functional activity of the *vapBC2* locus has been confirmed relatively recently [7,8].

The physiological effect of the toxin–antitoxin system *mazEF* in *M. smegmatis* has been observed to be realized through tRNA^Lys^ cleavage, resulting in proteome reprogramming. This reprogramming leads to the downregulation of proteins rich in lysine AAA codons and the upregulation of proteins deficient in lysine codons. Thus, under conditions of MazF expression in cells, there is a decrease in the synthesis of critical components of the DNA replication machinery and an increase in the translation of stress response proteins [9]. The *vapBC* system of *M. smegmatis* is involved in the regulation of translation by means of 23S rRNA cleavage [10], which is supposed to cause the deactivation of ribosomes and their deposition in a state associated with the membrane [11], ultimately leading to the transition of mycobacterial cells to a dormant state [12]. The physiological effects of *vapBC2* are associated with changes in sensitivity to certain antibiotics and oxidative stress, but the molecular mechanism of action of this TA system has not yet been described [7].

In general, the following functions are known for *vapBC* systems to date. They contribute to the resting state development [12], influence the bacterial symbiotic interaction with plant partners [13], and participate in *Salmonella typhimurium* persister formation in the course of infection of eukaryotic cells [14]. The demonstrated deceleration in protein synthesis under VapC expression and bacterial growth slowdown resultant from tRNA slicing reveals the toxin role as the accurate tuner of the translation process and contributes to stress response development, in particular, under treatment with antibiotics inhibiting ribosome activity [15]. In turn, the *mazEF* module is associated with the formation of persisting and dormant bacteria [16,17] and affects antibiotic resistance [18] as well as susceptibility to different environmental stresses [9,19].

In the current paper, we studied *vapBC* and *mazEF* TA systems’ functional features, though the *vapBC2* module had not been taken into account. Proteomic profiling data and the effects of VapC overexpression on the susceptibility of *M. smegmatis* cells to heat shock and antibiotic treatment were analyzed. In order to understand the mechanisms behind the altered stress responses in *M. smegmatis* cells, we conducted experiments using *vapBC*, *mazEF*, and double *vapBC-mazEF* deletion mutants. We measured the levels of transcription of the TA loci after supplementing with tetracycline as well. Our results indicate that both the *vapBC* and *mazEF* systems play a role in stress responses in *M. smegmatis*, and the fate of the cells depends on the interaction between these TA loci.

## 2. Materials and Methods

### 2.1. Bacterial Strains, Media, and Growth Conditions

All experiments were performed using *Mycobacterium smegmatis* strain mc^2^ 155 (ATCC 700084) and its derivatives that were obtained earlier [12] or constructed in the course of this research (see “DNA manipulation” section). While working with VapC overexpressing strain *M. smegmatis* mc^2^ 155 pMind_vapC (VapC over), plasmid-bearing *M. smegmatis* mc^2^ 155 pMind (wt + pMind) was used as the empty vector control strain. For all deletion mutants the wild-type strain (wt) was employed as the control.

All *M. smegmatis* strains used in the current work were aerobically cultured for 24–30 h at 37 °C on an orbital shaker with agitation (200 rpm) in tubes containing 4 mL of Nutrient Broth medium (Himedia, Mumbai, India). To obtain a homogeneous growing culture, Tween-80 was added to the medium at a concentration of 0.05%. The resulting inoculum was used as the material for further experimental work. When culturing strains obtained through pMind plasmid transformation, hygromycin B Gold (InvivoGen, San Diego, CA, USA) was added to the growth medium at a concentration of 50 µg/mL. VapC overexpression was induced by the addition of tetracycline (20 ng/mL).

### 2.2. DNA Manipulation

To create the *mazEF* deletion strain, we employed the commonly used two-step strategy. First, we amplified the upstream (L) and downstream (R) regions of the *mazEF* locus using the primer pairs UpMazLDel/LowMazLDel and UpMazRDel/LowMazRDel, respectively. The primer sequences can be found in Table A1. The obtained PCR products were then used as templates for overlap extension PCR with the UpMazLDel and LowMazRDel primers. The resulting 2126 bp amplicon was purified and digested with HindIII and BamHI restriction endonucleases. The sticky ends of the digested amplicon were cloned into the p2NIL vector. This resulted in the creation of the p2NIL-MazFDel vector, which was then ligated with the pGOAL19-PacI cassette. The resulting plasmid was purified and used in the construction of the deletion mutant, following the previously described method [20]. *M. smegmatis* cells were transformed with the vectors using electroporation. The resultant and intermediate plasmids, as well as the final deletion mutant strain, were verified using PCR and analyzed by Sanger sequencing. The same approach was used to construct the ΔvapBCΔmazEF double mutant, with the only difference being that the ΔvapBC cells were transformed with the p2NIL-MazFDel vector instead of the wild-type *M. smegmatis* culture.

### 2.3. Determination of the Minimum Inhibitory Concentrations

The working solutions of each antibiotic were prepared by making two-fold dilutions of the stock solutions in Nutrient broth (Himedia, Mumbai, India). *M. smegmatis* cultures of all studied strains were cultivated overnight under the conditions described above (refer to “Bacterial strains, media, and growth conditions” section) until they reached the mid-log phase of growth. The bacterial cultures were adjusted to optical density OD_600_ = 0.1 with the use of a spectrophotometer DiluPhotometer (Implen, München, Germany). A total of 100 µL of each antibiotic working solution was dispensed into separate wells of the 96-well microtiter plate (Corning Inc., Corning, NY, USA), starting from the highest concentration towards the lowest concentration. Then, 100 µL of the diluted bacterial cultures were added to each well, ensuring that the final volume was 200 µL per well and including positive and negative controls in the plate. The positive control contained only the bacterial culture without any antibiotic, whereas the negative control contained only the antibiotic-working solution without any bacteria. The plates were incubated at 37 °C for 72 h. After the incubation period, the wells were visually inspected for growth. The minimum inhibitory concentration (MIC) was defined as the lowest concentration of antibiotic that completely inhibited the visible growth of the bacteria. The experiment was repeated three times to ensure reproducibility.

### 2.4. Heat Shock, Antibiotic Treatment, MPN Test

The tolerance to heat shock was determined by comparing the colony-forming units (CFUs) after heating 1 mL aliquots of the studied cultures in the temperature range of 60 °C to 70 °C for 10 min. The proportion of antibiotic-resistant cells was determined by comparing the CFU values after a 24 h incubation of cells in the presence of ciprofloxacin (2.5 µg/mL), tetracycline (5 µg/mL), and isoniazid (100 µg/mL). Tetracycline treatment was also conducted for 7 days with plating the treated cells after 24 h, 48 h, and 7 days of antibiotic exposure. Prior to plating, the treated cells were washed with 50 mM phosphate buffer (pH 7.0). To determine the CFU values, cells were plated on Petri dishes containing solid Nutrient Broth medium (Himedia, Mumbai, India) supplemented with 1.5% agar.

The assessment of the cells’ viability was based on their ability to form colonies on solid nutrient media. A series of tenfold dilutions of the bacterial cell suspensions were prepared in fresh growth medium, and then 100 µL of each dilution was plated on agarized (1.5% agar) Nutrient Broth. The number of colony-forming units (CFU) was counted after 3 days of incubation and determined as the average of three replicates. The most probable number (MPN) test was performed in a 48-well plastic plate (CorningInc., Corning, NY, USA), with each well containing 0.9 mL of Nutrient broth medium. Each well also contained 0.1 mL of the corresponding serial dilutions of cells. Each dilution was represented in three replicates. The plates were incubated for 6–10 days at 37 °C on an orbital shaker (with 120 rpm agitation). When counting MPNs of resuscitated cells, wells with visible bacterial growth were taken into account. The estimated number of resuscitated cells per 1 mL was determined using standard statistical tables [21].

### 2.5. Protein Extraction, LC-MS/MS Analysis

Protein extraction and proteolytic in-gel digestion with trypsin (Trypsin Gold, Mass Spectrometry Grade, Promega, Madison, WI, USA) were performed as described previously [22].

LC-MS/MS analysis was conducted using a TripleTOF 5600+ mass-spectrometer equipped with a NanoSpray III ion source (AB Sciex, Concord, ON, Canada) connected to a NanoLC Ultra 2D+ nano-HPLC system (Eksigent, Singapore city, Singapore). The HPLC system was set up in a trap-elute mode. A mixture of 98.9% water, 1% methanol, and 0.1% formic acid (*v*/*v*) was used as the sample loading buffer and buffer A. Buffer B consisted of 99.9% acetonitrile and 0.1% formic acid (*v*/*v*). The samples were loaded onto a trap column Chrom XP C18, 3 mm, 120 A, 350 mm × 0.5 mm (Eksigent, Singapore) at a flow rate of 3.5 μL/min for 10 min and then eluted through the separation column 3C18-CL-120 (3 mm, 120 A) 75 mm × 150 mm (Eksigent, Singapore) at a flow rate of 300 nL/min. The gradient ranged from 5 to 40% of buffer B over a period of 120 min. The column and pre-column were cleaned between runs by washing with 95% buffer B for 7 min and equilibrated with 5% buffer B for 25 min. To prevent carryover, both the column and pre-column were thoroughly washed with a blank trap-elute gradient, including waves of buffer B ranging from 5-95% to 95-5% for 5–7 min followed by a 25 min equilibration with 5% buffer B. Mass spectra were acquired in the positive ion mode. Information-dependent mass-spectrometer experiments consisted of one survey MS1 scan followed by 50 dependent MS2 scans. The mass range for analysis and subsequent ion selection for MS2 analysis was 300–1250 *m*/*z*, with a signal accumulation time of 250 ms for MS1 acquisition. Ions for MS2 analysis were selected based on intensity, with a threshold of 200 cps and charge state between 2 and 5. For MS2 acquisition, the resolution of the quadrupole was set to UNIT (0.7 Da), the measurement mass range was 200–1800 *m*/*z*, and the ion beam focus was optimized to achieve maximum sensitivity. The signal accumulation time for each parent ion was 50 ms. Collision activated dissociation was performed using nitrogen gas, with collision energy ramping from 25 to 55 V within the 50 ms signal accumulation time. Analyzed parent ions were placed on a dynamic exclusion list for 15 s to collect the next MS2 spectra of the same compound around its chromatographic peak apex (the minimum peak width throughout the gradient was approximately 30 s).

### 2.6. Protein Identification and Quantitation

Raw data were captured from the mass spectrometer and converted to MGF (Mascot Generic Format) files using ProteoWizard with the following parameters: peakPicking true 2, msLevel 2, zeroSamples removeExtra [23]. For thorough protein identification, the generated peak lists were searched with the MASCOT (v 2.5.1, Matrix Science Ltd., London, UK) and X! Tandem (VENGEANCE, 2015.12.15, The Global Proteome Machine Organization) search engines. Database-searching parameters were as follows: tryptic hydrolysis, no more than one missed site, the precursor and fragment mass tolerance were set at 20 ppm and 50 ppm, respectively. Oxidation of methionine was set as a possible modification, carbamidomethylation of cysteine as a fixed. For X! Tandem, parameters that allowed a quick check for protein N-terminal residue acetylation, peptide N-terminal glutamine ammonia loss or peptide N-terminal glutamic acid water loss were selected. Resulting files were submitted to the Scaffold 4 software (v 4.2.1, Proteome Software, Inc., Portland, OR, USA) for validation and further analysis. For protein identification, the proteomic databases for the *M. smegmatis* mc^2^ 155 (RefSeq:NC_008596.1) genome were used. The local false discovery rate scoring algorithm with standard experiment-wide protein grouping was used. A 1% FDR threshold was applied to search results from individual datasets. For all detected proteins, functional categories (Mycobrowser v 3.0 (https://mycobrowser.epfl.ch/, accessed on 25 September 2023)) and subcellular localizations (PSORTdb v 4.0 (http://db.psort.org/, accessed on 25 September 2023), proteomic data were aligned to *M. tuberculosis* orthologs) were established.

For label-free quantitation, raw MS data files (.wiff files) were imported and processed in Progenesis LC-MS software v.4.1 (Nonlinear Dynamics, Newcastle, UK). The results of peptide quantitation were normalized using an iterative median-based normalization as implemented in the Progenesis software v.4.1. Differences in the abundance of a protein between the three biological replicates of all strains were evaluated using a two-sided unpaired Student’s *t*-test. *p*-values < 0.05 were considered statistically significant. Adjusted *p*-values for multiple tests (q-values) were generated using the Benjamini–Hochberg method [24].

### 2.7. Isolation of RNA, RNA Electrophoresis, qRT-PCR

Samples of total RNA were obtained by phenol-chloroform extraction as described previously [25]. Following isolation, RNA extracts were treated with Turbo DNase (Life Technologies, Carlsbad, CA, USA) to eliminate any genomic DNA remnants. The purified RNA was then visualized using a 1% agarose gel in TAE buffer. For cDNA synthesis, 100 ng of the extracted RNA was utilized along with random hexanucleotides and SuperScript III reverse transcriptase (ThermoFisher Scientific, Waltham, MA, USA). To conduct qPCR, the qPCRmix-HS SYBR (Evrogen, Moscow, Russia) was employed, along with specific primers including maz_RT_F, maz_RT_R, vapC_RT_F, vapC_RT_R, 16SMSM_RTUp, and 16SMSM_RTLow (refer to Table A1 for primer sequences). The Applied Biosystems 7300 Real-Time PCR system (ThermoFisher Scientific, USA) was used for qPCR, with the following cycling conditions: 95 °C for 10 s, 60 °C for 10 s, and 72 °C for 30 s, repeated for 40 cycles. LinRegPCR v. 11.0 (the Academic Medical Center, Amsterdam, The Netherlands) was employed to process the PCR data. The abundance of amplicons was calculated relative to the expression level of the 16S rRNA gene.

### 2.8. Statistical Analysis

The results of physiological studies were obtained in at least three replicates and processed with the use of STATISTICA v.7.0 software (StatSoft, Tulsa, OK, USA). To determine the significance of differences, Student’s *t*-test adjusted for multiple comparisons was used at the accepted significance level of *p* ≤ 0.05.

## 3. Results

### 3.1. VapC Overexpression Leads to Proteomic Changes in Abundance of Proteins Involved in Primary Metabolism and Stress Response

In a previous study, we demonstrated the 23S rRNA cleavage when VapC was overexpressed in *M. smegmatis*. Additionally, we observed significant alterations in the components of the protein-synthesis machinery under these conditions [11]. In the current work, we analyzed the substantial modifications in the proteins involved in metabolic processes and stress responses according to the proteomic profiling data obtained earlier in *M. smegmatis* cultures overexpressing VapC (please refer to the data availability statement to find the deposited data).

During the process of quantitative proteome analysis, we specifically chose distinct polypeptides that were detected in all the samples examined. Only those polypeptides that exhibited an increase in at least two-fold or a decrease in more than two-fold under VapC overexpression, in comparison to the control wild-type samples, were considered for further investigation.

**Figure A1:**
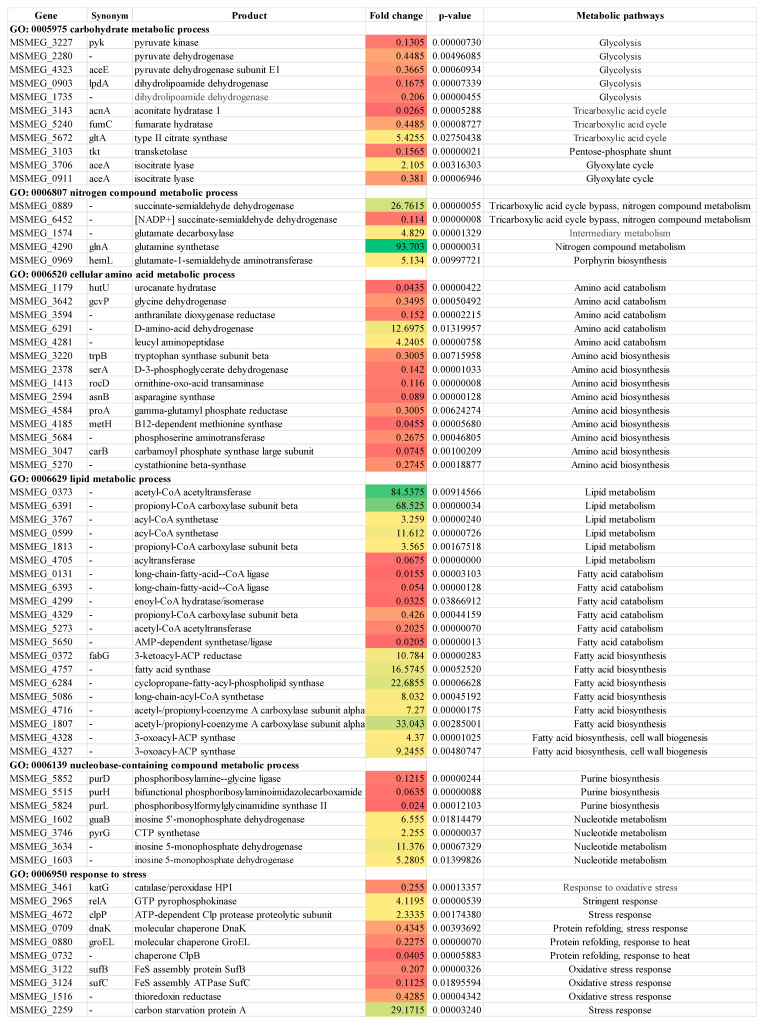
Functional clusters of differential proteins discussed in the text. The proteins with decreased abundance are highlighted with shades of red, while the proteins with increased abundance are highlighted with shades of green, according to the fold change. The main gene ontology (GO) categories are given in bold.

A detailed analysis of proteomic data allowed us to observe certain changes in primary metabolic and regulatory processes under VapC overexpression (see Figure A1). Specifically, a decrease in abundance was detected for a number of enzymes involved in glycolysis (pyruvate kinase MSMEG_3227, pyruvate dehydrogenase subunits MSMEG_2280, MSMEG_4323, MSMEG_0903), tricarboxylic acid cycle (e.g., aconitate hydratase MSMEG_3143, fumarate hydratase MSMEG_5240), and pentose-phosphate shunt (transketolase MSMEG_3103) of carbohydrate metabolism. At the same time, the abundance of citrate synthase MSMEG_5672, which is involved in glyoxylate shunt, was increased, whereas isocitrate lyase MSMEG_0911 was replaced by isoenzyme MSMEG_3706. A similar phenomenon was observed for gamma-aminobutyric acid shunt, i.e., succinate-semialdehyde dehydrogenase MSMEG_6452 was substituted by isoenzyme MSMEG_0889.

The data on the abundance of proteins involved in amino acid metabolism under VapC overexpression demonstrated the decrease amidst synthase enzymes, such as tryptophan synthase MSMEG_3220, asparagine synthase MSMEG_2594, methionine synthase MSMEG_4185, carbamoyl phosphate synthase MSMEG_3047, etc. (nine enzymes overall). However, the abundance ratio of glutamine synthetase MSMEG_4290 was 94-fold higher when compared to the control. Moreover, three enzymes of amino acid catabolism were identified as under-represented proteins, and two more proteins were over-represented in this category (refer to Figure A1).

VapC overexpression also affected the abundance of a number of enzymes participating in fatty acid biosynthesis as most of them were over-represented, whereas certain enzymes attributed to fatty acid degradation pathways were under-represented to a large extent. Acetyl-CoA acetyltransferase MSMEG_5273 was substituted by isoenzyme MSMEG_0373 following VapC overexpression, and its abundance ratio was nearly 85-fold higher in comparison with the control strain (Figure A1).

Furthermore, we detected three enzymes encoded by *pur* genes to be under-represented according to our proteomic data, which implied that nucleotide metabolism could have been affected by VapC overexpression as well. At the same time, three isoforms of inosine 5-monophosphate dehydrogenase were identified as over-represented. A notable find was the increased abundance of HemL protein (MSMEG_0969) which is involved in tetrapyrrole biosynthesis and thus participates in porphyrin production (Figure A1).

Simultaneously, significant changes in the abundance of stress response proteins were observed under VapC overexpression. In this group, carbon starvation protein A (MSMEG_2259) was the most over-represented (a 29-fold increase in abundance in comparison with the control), it was followed by GTP pyrophosphokinase RelA (MSMEG_2965) with a fourfold increase in abundance relative to the control. Some polypeptides associated with oxidative stress response, e.g., catalase MSMEG_3461, FeS assembly proteins MSMEG_3122, MSMEG_3124, as well as thioredoxin reductase MSMEG_1516, were identified as under-represented. Another finding worth mentioning was the replacement of various proteins participating in oxidative stress response in our proteomic data; for instance, Mn-superoxide dismutase (MSMEG_6636) was replaced by copper/zinc superoxide dismutase (MSMEG_0835) and KatG catalase/peroxidase HPI MSMEG_3461 was substituted by isoenzyme MSMEG_6384.

Finally, proteomic profiling of VapC overexpressing samples demonstrated an increased abundance of ClpP protease (MSMEG_4672), accompanied by a decreased abundance of GroEL chaperone (MSMEG_0880) and chaperon DnaK (MSMEG_0709), whereas heat shock proteins, such as ClpX protease subunit (MSMEG_4671) and molecular chaperone DnaJ (MSMEG_0711, MSMEG_4504), were identified as unique, i.e., they were detected only in proteomic profiles of VapC overexpressing strain though not in the control ones.

### 3.2. VapC Influences M. smegmatis Susceptibility to Some Stressful Conditions

The observed proteomic rearrangements associated with the abundance of proteins involved in the primary metabolic processes as well as in the adaptation to stressful conditions should also lead to phenotypic manifestations at the level of bacterial physiology. Therefore, we conducted a study on the resistance of *M. smegmatis* strains with overexpression of the VapC toxin, as well as with the deletion of the *vapBC* locus, to heat shock and antibiotic treatment.

Preliminary determination of the minimum inhibitory concentrations (MICs) of several antibiotics (tetracycline, ciprofloxacin, isoniazid) was conducted for all the recombinant strains of *M. smegmatis* included in this study and obtained during the course of this research (Table 1). Based on the results of this determination, no differences in the minimum inhibitory concentrations were found for exponential cultures of the investigated strains. Therefore, a single inhibitory concentration of each antibiotic was used to evaluate the resistance of *M. smegmatis* strains with overexpression and deletions of target toxins, and it was greater than the minimum inhibitory concentration by at least 10 times.

**Figure 1:**
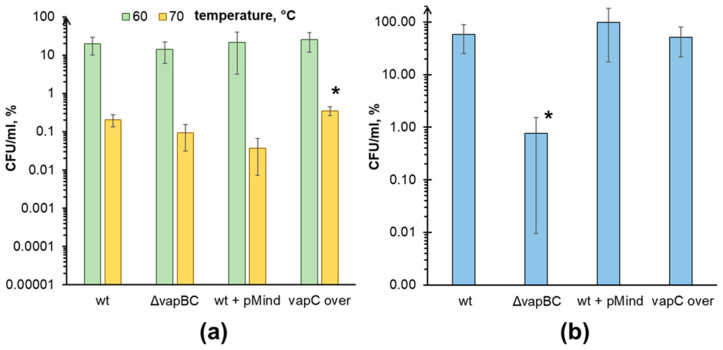
VapC toxin is involved in *M. smegmatis* stress susceptibility. Suspensions of 48 h *M. smegmatis* cells of the wild type, ΔvapBC, and wild-type cells transformed with the empty pMind vector (wt + pMind), and VapC overexpressing (vapC over) cells were subjected to a 10 min heat treatment at 60 and 70 °C (**a**) or treated with 5 µg/mL tetracycline for 24 h (**b**). Following antibiotic treatment, bacterial cells were washed with saline and plated into Petri dishes. Data are represented in relative CFU/mL units (%), which were calculated with respect to CFU/mL numbers in the untreated cultures at the starting time point. Mean values were calculated out of at least 3 independent experiments and are represented with standard deviation margins. An asterisk (*) indicates statistically significant differences with the corresponding control culture (see bacterial strains, media, and growth conditions subsection in the Materials and Methods section).

Our findings revealed that *M. smegmatis* cells with induced VapC toxin production exhibited increased resistance to heat shock at 70 °C (0.354% viable cells) when compared to the empty vector control culture (0.0373% viable cells). In contrast, the ΔvapBC strain demonstrated decreased resistance to higher temperatures when compared to the wild type (0.0939% vs 0.207% viable cells, respectively) under the conditions studied (Figure 1a).

We observed no difference in the viability of *M. smegmatis* VapC overexpressing and ΔvapBC strains compared to their corresponding control cultures when treated with ciprofloxacin and isoniazid, as measured by relative CFU numbers. However, after 24 h of tetracycline supplementation, a significant decrease in CFU number (two orders of magnitude) was observed in the ΔvapBC strain (Figure 1b). It should be noted that no changes in viability were found in the VapC overexpressing culture, suggesting that the antibiotic effect was likely indirect or influenced by another factor. Since there is a *mazEF* TA system in *M. smegmatis* that could also be involved in the response to antibiotic stress, we investigated the effect of tetracycline on a ΔmazEF strain to further explore this assumption.

### 3.3. vapBC and mazEF TA Systems of M. smegmatis Show Different Effects under Tetracycline Treatment

**Figure 2:**
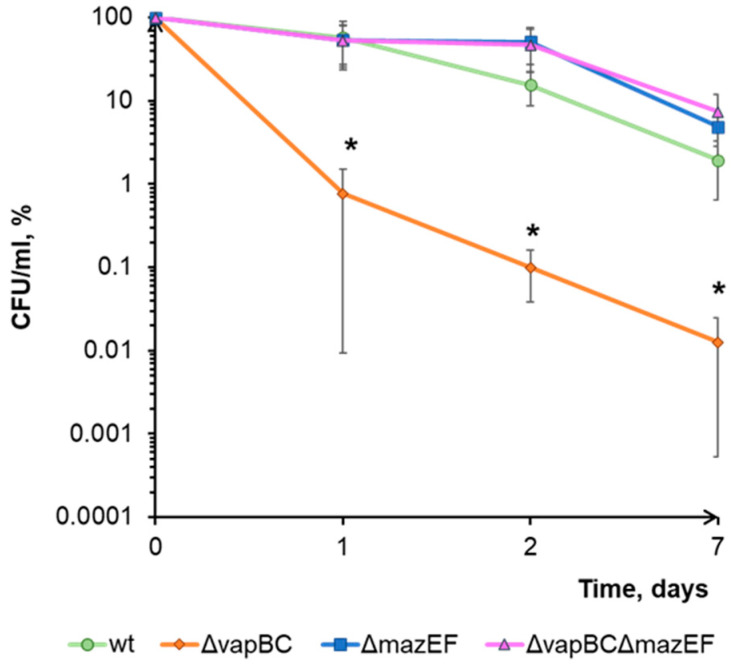
Both VapBC and MazEF TA systems are involved in *M. smegmatis* viability under tetracycline treatment. Suspensions of 48 h cells of the wild type, ΔvapBC, ΔmazEF, and ΔvapBCΔmazEF strains of *M. smegmatis* were treated with 5 µg/mL tetracycline. Following the antibiotic treatment, bacterial cells were washed with saline and plated into Petri dishes. Data are represented in relative CFU/mL (%), which were calculated with respect to CFU numbers in the untreated cultures at the starting time point. Mean values were calculated out of at least 3 independent experiments and are represented with standard deviation margins. An asterisk (*) indicates statistically significant differences with the corresponding control culture.

After 24 h of exposure to tetracycline, no effect of the *mazEF* deletion on the viability of the *M. smegmatis* was observed (Figure 2). At the same time, the double deletion strain (ΔvapBCΔmazEF) developed a restoration of the wild-type phenotype as the culture death observed with *vapBC* deletion was abolished. After 48 h of tetracycline exposure, the significance of the MazEF system was pronounced even more, i.e., ΔvapBCΔmazEF cultures showed not only a reversion of the ΔvapBC phenotype to the wild type but also a higher survival rate of bacterial cultures in the presence of this antibiotic (Figure 2, Table 2). A similar level of CFU value was observed in the ΔmazEF strain.

Mycobacteria are known to develop a dormant state that is characterized by the ability of cells to remain alive but not to form colonies on solid media, so the growth of colonies on Petri dishes does not always reflect the true survival rate of the bacteria. The ratio of the dormant cells in the population can be measured by comparing CFU values with the results of the most probable number (MPN) test [26]. After conducting such an analysis, we found that the CFU decrease in ΔvapBC strain under tetracycline treatment was partially associated with cell transition to an unculturable dormant state, although the survival rate recession was still caused mostly by cell death (Table 2). It is important to note that in the ΔvapBCΔmazEF strain, the percentage of dormant cells was reversed to the level of the wild-type strain, although the absolute values of viability were almost threefold higher than in the control.

In many cases, complementation of a deleted locus is important for confirming the restoration of the wild-type phenotype. However, in the case of toxin–antitoxin loci, this is not a trivial task, as in the wild type, the expression of toxins and antitoxins is driven by their own promoter, and the adjustment of expression may occur stochastically and be coupled with post-translational regulation, particularly with proteolysis of antitoxin molecules. In the case of complementing deleted TA loci by introducing plasmid DNA bearing the entire locus, the toxin and antitoxin genes are expressed not from a native promoter, which leads, generally, to the formation of cells with a phenotype different from the wild type in terms of physiological state. In our previous experiments, we overexpressed entire TA loci, which did not lead to any changes, probably due to the fact that under conditions of artificial overexpression, toxins and antitoxins neutralize each other. Other researchers have also induced overexpression of several *M. smegmatis* TA loci individually, and all resulting recombinant strains, in particular, had the same growth rate [6]. Overall, complementation of deletions was not justified within the scope of this study.

### 3.4. vapC and mazF Expression Levels Are Increased under Tetracycline Treatment

Typically, cell defense systems are activated in response to a particular stress. At the same time, bacteria are able to develop a general stress response that allows cells to pre-adapt to a wide range of stressful conditions, regardless of the nature of the initial stress factor [27]. Due to their growth arrest and decreased metabolism rate, dormant bacteria can often be tolerant to various stressful conditions, including antibiotics [28], thus demonstrating general stress resistance. Bacterial dormancy may be considered as a mechanistic basis for persistence [29], and persistent bacteria, in turn, may be formed either spontaneously or following triggering by a specific stress signal [30]. From here, and taking into account that VapC and MazF toxins were shown to contribute to altered tetracycline susceptibility, we studied if their expression rate could be affected by tetracycline treatment.

**Figure 3:**
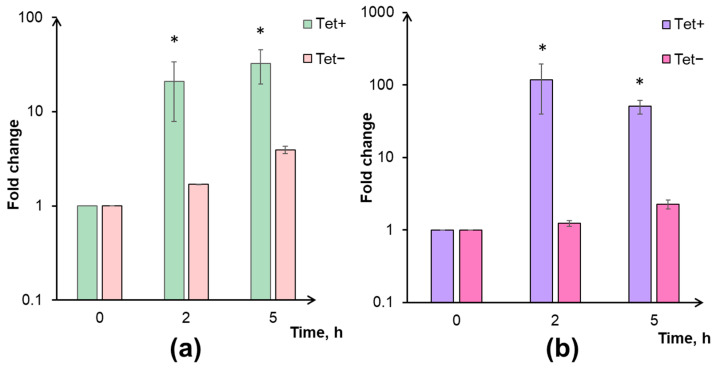
The results of qRT-PCR analysis performed in M. smegmatis samples treated with 5 µg/mL tetracycline (Tet+) in comparison with untreated control cultures (Tet−). The mRNA expression levels of vapC (**a**) and mazF (**b**) were calculated relative to 16S mRNA transcripts and presented as fold change in mean ± SD format. An asterisk (*) indicates statistically significant differences with the point of 0 h.

Using the qRT-PCR technique, we observed that the expression rate of the *vapC* gene increased by approximately 21-fold after 2 h of incubation with tetracycline. At the same time, the expression rate of the *mazF* gene increased by nearly 117-fold (Figure 3a,b). This fact showed the inducible nature of VapC and MazF toxin expression levels increase following tetracycline supplementation.

## 4. Discussion

Proteomic profiling is a valuable tool that can assist in predicting the physiological characteristics of bacterial cells under specific conditions. In this study, we examined the differential proteins in cultures of *M. smegmatis* with overexpression of VapC compared to the wild-type strain. We then compared these proteins with the known features of bacterial stress responses.

Specifically, we observed a decrease in the abundance of carbohydrate metabolism proteins, such as pyruvate kinase, pyruvate dehydrogenase, aconitate hydratase, and fumarate hydratase, in addition to an increase in citrate synthase representation. Some enzymes were also substituted with their isoforms, such as isocitrate lyase and succinate-semialdehyde dehydrogenase. These changes suggest the activation of the glyoxylate shunt and 4-aminobutyric acid shunt, (which are tricarboxylic acid cycle bypass pathways), indicating that the cells are preparing for a stationary phase and potentially entering a dormant state [31,32]. Furthermore, we observed an increase in the abundance of proteins involved in fatty acid biosynthesis (at least eight over-represented proteins) and a decrease in enzymes involved in fatty acid catabolism (six under-represented proteins). This suggests that the bacteria may be accumulating fatty acids in the form of triacyl glycerides to counteract stressful conditions such as hypoxia and nutrient starvation, which are often encountered during stationary phase and dormancy development [33,34]. Additionally, we observed an increase in the abundance of HemL protein, which participates in porphyrin biosynthesis. Porphyrin accumulation is a characteristic of dormant cells in the case of *M. smegmatis* [35], further supporting the assumption that the cells are transitioning to a stationary phase and developing dormancy. On the other hand, the transition to the stationary phase and resting state development is usually associated with the activation of a general stress response [36]. Interestingly, overexpression of the VapC toxin led to similar changes in the proteomic data. We observed an increase in the abundance of the ClpP protease subunit, accompanied by the detection of the ClpX subunit as a unique protein. This suggests the activation of proteolysis, the breakdown of unfolded and misfolded proteins, and polypeptides with aborted synthesis [37]. However, microbial cells typically try to refold injured and denatured proteins before resorting to proteolysis. Therefore, changes in chaperone abundance would be expected under VapC overexpression. Indeed, we identified the molecular chaperone DnaJ, which can effectively restore aggregated and misfolded proteins independently of ATP availability [38], potentially replacing the decreased levels of DnaK and GroEL chaperones [39,40] under VapC overexpression.

Furthermore, it is commonly observed that stress conditions and the transition to the stationary phase result in a reduction in metabolic activity and energy production. This notion is supported by our findings, as we observed a downregulation of carbohydrate metabolism enzymes in response to these conditions. This possible decrease in metabolic activity may also be connected with a reduced respiration rate and decreased electron flow in the respiratory chain. Since the respiratory chain is a major source of reactive oxygen species in cells [41], the endogenous oxidative stress is reduced under these conditions [42]. Consistent with this, we found that oxidative stress-related proteins, such as thioredoxin reductase, which participates in antioxidant defense through the conversion of thiol and disulfide bonds [43], and SufBCD complex proteins, which are the components of the Suf machinery that is responsible for de novo iron-sulfur (Fe-S) cluster biogenesis [44], were less abundant under VapC overexpression.

In contrast, the Mn-superoxide dismutase SodA was replaced by the copper/zinc superoxide dismutase SodC, which is an enzyme induced during the stationary phase and dependent on the SigB factor. This enzyme provides protection against reactive oxygen intermediates at the surface of mycobacteria [45]. Additionally, the catalase KatG was substituted with an alternative isoenzyme, possibly similar to the stationary phase KatE of *E. coli* which is known to be less efficient though better fitting for stressful environments [46,47]. These two substitutions (SodA and KatG) may indicate a shift in the oxidative defense system of mycobacteria from internal to external challenges, similar to the oxidative stress experienced by cells within macrophages in the case of MTB or BCG infection [45,48,49]. 

Although the (p)ppGpp-synthase RelA was found to be highly expressed under VapC overexpression, the observed features of the stringent response were atypical. Contrary to the presumable increase in amino acid synthesis [50], we actually observed a decrease in the abundance of amino acid synthetase enzymes. Nonetheless, this decrease aligns with the overall reduction in synthetic processes during the transition to stationary phase and bacterial dormancy [36,51]. Additionally, we found that the abundance of proteins involved in purine nucleotide metabolism was altered, which also happens under enhanced (p)ppGpp production [52].

Surprisingly, we also observed an increase in the abundance of glutamine synthetase MSMEG_4290, which catalyzes the conversion of glutamate to glutamine. This contradicted our initial hypothesis that it would be repressed along with other amino acid synthetases. The physiological significance of this increase may be related to ammonium fixation [53] or, more probably, the utilization of amino groups following protein degradation [54] due to the premature release of incompletely synthesized polypeptide molecules out of the ribosome under aminoacyl-tRNA deficiency [55]. The synthesized glutamine may also play a role in the conversion of 2-ketoglutarate to glutamate, a key step in the 4-aminobutyric acid pathway (we also detected the increased abundance of succinate-semialdehyde dehydrogenase MSMEG_0889 that is the component of this pathway) [56]. Additionally, the upregulation of glutamate decarboxylase MSMEG_1574 suggests a potential role in acid stress resistance [57].

Overall, our proteomic profiling revealed changes indicative of a transition to alternative energy production processes, activation of emergency-associated biochemical pathways, and a general stress response. These proteomic features likely alter the physiology of mycobacterial cells.

The conducted investigation showed that VapC indeed participates in the regulation of bacterial sensitivity to high temperatures (Figure 1a). Activation of proteases and changes in the representation of chaperones observed under conditions of VapC overexpression are well consistent with increased heat resistance. Accordingly, the ΔvapBC strain demonstrates an increasing sensitivity to this adverse factor. Although the proteomic alterations observed in *M. smegmatis* cells upon VapC overexpression did not confer any protective advantage against the effects of tetracycline, the role of VapC was clearly pronounced upon deletion of the *vapBC* locus (Figure 1b). At the same time, the VapBC TA system did not affect the ability of mycobacterial cells to adapt to the action of ciprofloxacin and isoniazid. This is most likely related to the mechanism of action of antibiotics. Ciprofloxacin belongs to fluoroquinolones and is aimed at inhibiting DNA gyrase [58]. Isoniazid affects cell wall biogenesis by inhibiting enoyl reductase involved in the synthesis of mycolic acids [59]. In turn, tetracycline suppresses protein synthesis on the ribosome, preventing the interaction of aminoacyl-tRNA with the A-site of its 30S subunit [60]. Thus, ciprofloxacin suppresses replication, isoniazid suppresses cell wall synthesis, and tetracycline suppresses translation. The mechanism of action of VapC toxin, at least partially, is realized by the cleavage of 23S rRNA, which can lead to the removal of ribosomes from active protein synthesis, their deposition in a membrane-associated state, and the transition of bacterial cells into dormancy. Thus, VapC, like tetracycline, affects the translation apparatus, stopping its functioning, but ensuring preservation in an inactivated state [11]. This could potentially reduce the effectiveness of antibiotic action due to the partial vanishing of the target and the transition of cells into a dormant state; however, the absence of a positive effect of VapC overexpression on the survival of *M. smegmatis* (Figure 1b) raised doubt about this hypothesis. 

The situation became somewhat clearer when we evaluated the effect of tetracycline on the survival of the ΔmazEF and the double deletion ΔvapBCΔmazEF strains. Deletion of the second TA system in the ΔvapBC strain led not only to the cancellation of cell death but also to higher levels of *M. smegmatis* survival compared to the wild-type cells (Figure 2). A similar level of CFU was observed in the ΔmazEF mutant. Apparently, the decrease in viability of the ΔvapBC strain under the action of tetracycline was largely due to the activity of the MazF ribonuclease, which cleaves tRNA^Lys^ [9]. In wild-type cells, because of noncognate toxin–antitoxin interaction [61], MazF activity was partially blocked by the antitoxin VapB. In the ΔvapBC mutant, such interaction was impossible due to the absence of the VapB antitoxin, which led to a pronounced (two orders of magnitude) decrease in CFU value caused by MazF activity. Contrarily, the ΔmazEF and ΔVapBCΔmazEF strains lacked the MazF toxin in their cells and thus exhibited viability levels three times higher. Evaluation of *M. smegmatis* survival using the most probable number (MPN) method showed similar patterns to CFU counting. At the same time, a comparison of CFU and MPN (Table 2) revealed the highest number of dormant cells in the ΔvapBC mutant, which likely indicates the involvement of the MazF toxin in the formation of such forms under the action of tetracycline. It should be noted that the observed maximum proportion of non-culturable bacterial forms in the ΔvapBC strain does not exclude the possibility of VapC toxin’s involvement in the transition to a dormant state but suggests the existence of a complex ensemble of interacting molecules in which VapC and MazF play noticeable roles. The phenotype reversion to the wild-type MPN levels in double deletion mutant (ΔvapBCΔmazEF strain) supports the idea of the TA systems interaction (Table 2). It is likely that each of the VapBC and MazEF modules acts as a significant factor in the formation of dormant cells in certain specific environmental conditions.

Indirect confirmation of this assumption may be detecting the activation of *vapC* and *mazF* gene expression under the action of tetracycline (Figure 3). The increase in the content of corresponding proteins in cells, on the one hand, contributes to the development of a general stress response and leads to the formation of a pool of dormant cells that are resistant to the action of the antibiotic. On the other hand, this leads to the death of part of the bacterial population through a mechanism not directly related to the action of the antibiotic but due to the activity of the MazF toxin. The fate of a specific cell, in this case, apparently, depends on the ratio of toxin content in it. From this perspective, the absence of a positive effect of VapC overexpression on survival under the action of tetracycline (Figure 1b) can be explained as follows. The artificially elevated concentration of the toxin contributes to slowing down growth and transitioning part of the population into a dormant state [11,12]. When tetracycline is added, these dormant cells become less sensitive to the antibiotic. In active cells, the induction of vapBC and mazEF expression occurs, during which the synthesized MazF toxin is partially blocked by the antitoxin VapB. At the same time, a portion of VapB molecules is associated with VapC due to its artificially increased level. Consequently, the pool of VapB protein, which is able to bind MazF toxin, is restricted allowing MazF to cleave tRNA^Lys^ and influence cell viability. Thus, the final CFU level becomes comparable to the survival in the wild-type culture, with the difference being that the strain with VapC overexpression is characterized by a slightly higher proportion of non-culturable cells and greater MazF activity, which leads to lower CFU levels.

## 5. Conclusions

Based on proteomic profiling, it can be concluded that overexpression of VapC in an *M. smegmatis* culture causes metabolic processes to be redirected and activates a general stress response, resulting in decreased sensitivity to high temperatures. The VapBC TA module does not affect susceptibility to replication inhibitors (such as ciprofloxacin) and cell wall synthesis inhibitors (such as isoniazid) during antibiotic treatment. However, it does play a role in evading the effects of ribosome-targeting antibiotics such as tetracycline. This could be achieved through the joint induction of the *vapBC* and *mazEF* TA modules, where MazF kills bacterial cells and VapBC can deactivate the toxin through non-cognate toxin–antitoxin interactions (specifically MazF-VapB interactions). The fate of the cell depends on the interplay between these TA modules, at least partially. These findings have potential implications for the development of new drugs. One possibility is to enhance the effectiveness of existing antibacterials by activating the MazF toxin. Another option is to reduce the expression level of *vapBC*, which could help prevent microbial tolerance to antibiotic treatment. It is worth noting that the conditions under which TA systems are upregulated may be more diverse than what we have observed in this study. Additionally, further research is needed to understand their role in the formation of dormant bacterial cells and their ability to withstand different environmental challenges.

## Figures and Tables

**Table 1:** Minimum inhibitory concentrations of three antibiotics for all *M. smegmatis* strains included in this research.

Strain	Minimum Inhibitory Concentration, µg/mL		
	Tetracycline	Ciprofloxacin	Isoniazid
wt	0.313	0.250	0.940
ΔvapBC	0.313	0.250	0.940
ΔmazEF	0.313	0.250	0.940
ΔvapBCΔmazEF	0.313	0.250	0.940
wt + pMind	0.313	0.250	0.940
vapC over	0.313	0.250	0.940

**Table 2:** Comparison of CFU and MPN values at the 48 h point of tetracycline (5 µg/mL) exposure. Statistically significant differences relative to the control (wt) are indicated in *italics*. Statistically significant differences between CFU and MPN values are indicated in **bold**.

Strain	CFU, %	MPN, %	CFU/MPN
wt	15.384 ± 6.747	22.9 ± 4.351 × 10^−15^	1.489
ΔvapBC	*0.099 ± 0.060*	*1.548 ± 0.701*	**15.635**
ΔmazEF	51.167 ± 24.197	43.75 ± 10.825	0.855
ΔvapBCΔmazEF	47.012 ± 4.530	72.9 ± 26.443	1.551

## Data Availability

All data associated with the current study are available on request from the authors. More details on proteomic profiling results can be found and downloaded at https://doi.org/10.1007/s00203-022-03363-1 [11].

## Appendix A

**Table A1:** Primers used in the current work.

Name	Sequence	Direction
UpMazLDel	AGAAGCTTCCGCGGATGCTGCCCAACGAAGAC	Forward
LowMazLDel	GAGTGCGCGGCCGGCGAAATTCGGGTGCATTCA	Reverse
UpMazRDel	ATTTCGCCGGCCGCGCACTCCTGGTTTTTCT	Forward
LowMazRDel	CAGGATCCACGATGGGCGGATTGTTGACG	Reverse
maz_RT_F	CTCATCATCCAGGACGACCGA	Forward
maz_RT_R	GCGTTGGCTCGATTCGAATT	Reverse
vapC_RT_F	GACACTTCTGCCCTCGTTGCCA	Forward
vapC_RT_R	CAGATACGACGCGGTGGACAT	Reverse
16SMSM_RTUp	GCAGGTCTCTGGGCTGTAAC	Forward
16SMSM_RTLow	CGTTTACGGCATGGACTACC	Reverse
